# Multi-Level Analog Resistive Switching Characteristics in Tri-Layer HfO_2_/Al_2_O_3_/HfO_2_ Based Memristor on ITO Electrode

**DOI:** 10.3390/nano10102069

**Published:** 2020-10-20

**Authors:** Chandreswar Mahata, Myounggon Kang, Sungjun Kim

**Affiliations:** 1 School of Electronics Engineering, Chungbuk National University, Cheongju 28644, Korea; chandreswar@gmail.com; 2 Department of Electronics Engineering, Korea National University of Transportation, Chungju-si 27469, Korea; 3 Division of Electronics and Electrical Engineering, Dongguk University, Seoul 04620, Korea

**Keywords:** HfO_2_/Al_2_O_3_/HfO_2_ tri-layer RRAM, transparent electrode, multilevel conductance, synaptic properties

## Abstract

Atomic layer deposited (ALD) HfO_2_/Al_2_O_3_/HfO_2_ tri-layer resistive random access memory (RRAM) structure has been studied with a transparent indium tin oxide (ITO) transparent electrode. Highly stable and reliable multilevel conductance can be controlled by the set current compliance and reset stop voltage in bipolar resistive switching. Improved gradual resistive switching was achieved because of the interdiffusion in the HfO_2_/Al_2_O_3_ interface where tri-valent Al incorporates with HfO_2_ and produces HfAlO. The uniformity in bipolar resistive switching with I_on_/I_off_ ratio (>10) and excellent endurance up to >10^3^ cycles was achieved. Multilevel conductance levels in potentiation/depression were realized with constant amplitude pulse train and increasing pulse amplitude. Thus, tri-layer structure-based RRAM can be a potential candidate for the synaptic device in neuromorphic computing.

## 1. Introduction

The physical limitation of the conventional flash memory gives rise to the development of resistive random access memory (RRAM) due to its low power consumption, higher density, and simple structure, which consist of mainly transition metal oxides sandwiched between the top and bottom electrodes [1,2,3,4,5,6]. Although, single layer RRAM devices have been found to have uncontrolled filament formation and high switching voltage [7,8,9]. Therefore, research has been focused on gaining low-power RRAM devices for symmetric SET/RESET behavior, gradual conductance change, and high synaptic density. Transition metal oxide, mainly based on Al_2_O_3_, HfO_2_, TaO_x_, ZrO_2_, TiO_2,_ and their bilayer and tri-layer structure, was shown to be advantageous for industry-friendly electrical devices and improved multilevel resistive switching properties and its application towards synapses for neuromorphic computing [10,11,12,13,14,15,16]. 

Bilayer RRAM structures already have shown both abrupt and gradual change in conductance under bipolar resistive switching, which has been proposed effective for neuromorphic computing with tunable potentiation and depression. Among different bilayer structures, recently Al_2_O_3_/Ta_2_O_5_, HfO_x_/HfO_2_, HfO_2_/TiO_x_, TaO_x_/Al_2_O_3_, Al_2_O_3_/TiO_2_ have been proposed to modulate the RRAM conductance gradually during resistive switching [5,13,17,18,19]. In bilayer structures mostly, oxygen vacancies were found to be located at the bilayer interface, thus the push and pull of oxygen vacancies from the interface mainly controls the resistance of the device during switching. Additionally, in other devices, modulation of the conductance can be controlled by the motion of oxygen vacancies between oxygen-deficient and oxygen-rich layers, which further limits the device’s operation current and controls low power consumption in RRAM. On the other hand, related to the multi-layer resistive switching, Wang et al., described the tri-layer RRAM structure of Al_2_O_3_/HfO_2_/Al_2_O_3_ with abrupt resistive switching performance [20]. In this work, it is considered that the concentration of oxygen vacancy is higher in HfO_2_ compared to Al_2_O_3_ and interfacial diffusion took place between two dielectrics. Therefore, the interfacial layer helps to exchange oxygen vacancies (V_O_), which finally improves resistive switching. A similar phenomenon has been indicated by Lui et al., where multilevel conductance was modulated by V_Reset-stop_ in Al_2_O_3_/HfO_2_/Al_2_O_3_ RRAM structure [21]. In the case of unipolar resistive switching, an important finding was explained by Maestro-Izquierdo et al., where 3D simulation suggests that during the RESET process, temperature distributions are different in multilayer structures [22]. Abrupt switching took place due to conductive filament (CF) narrowing in the HfO_2_ middle layer due to lower thermal conductivity of HfO_2_ (1.0 W m^−1^ K^−1^) compared to Al_2_O_3_ (2.86 W m^−1^ K^−1^) [23]. Furthermore, the tri-layer formed by inserting an oxygen-deficient ZrO_2‒x_ layer between two ZrO_2_ dielectric layers was found to have transitioned from interfacial to filamentary switching characteristics under different SET compliance currents [24]. However, it has previously been reported that the V_O_ from CF has low mobility in the AlO_x_ layer compared to HfO_2_ during the reset operation [25]. As compared to other different tri-layer RRAM structures mentioned above, for tri-layer RRAM structure in this study, the Al_2_O_3_ layer is placed between two higher Vo contents in HfO_2_ layers for better movement of the Vo during SET and RESET operations. In addition, the virtual electrode formed during the electroforming process within Vo rich HfO_2_ layers at both ends can help the CF regrowth during SET operation at lower electric field [26].

For the next generation of electronic devices, transparent electronics are recently emerging [27,28,29]. The development of transparent electronics including touch panel, display, energy storage, photodetector, and solar cells have attracted great interest. RRAM on transparent electrodes such as indium tin oxide (ITO), with gradual multilevel resistive switching for logic and memory devices, has gained increasing attention as a reliable synaptic device. Hence, it is highly expected that future RRAM embedded with transparent electrode (ITO) will become a paradigm for future see-through memory devices.

In this work, the proposed atomic layer deposited (ALD) HfO_2_/Al_2_O_3_/HfO_2_ tri-layer RRAM structure with a transparent ITO electrode has been studied in detail, which is believed to be more suitable for multilevel resistive switching. In addition, considering HfO_2_ has larger oxygen vacancies compared to Al_2_O_3_, this structure has been designed to have two interlayers formed between the top and bottom HfO_2_ layers with a middle Al_2_O_3_ layer. Excellent multi-resistance states were achieved for tri-layer RRAM to emulate neuromorphic properties. For application in synaptic devices, multilevel conductance was achieved by applying both DC and pulse voltages. 

## 2. Materials and Methods

**Figure 1:**
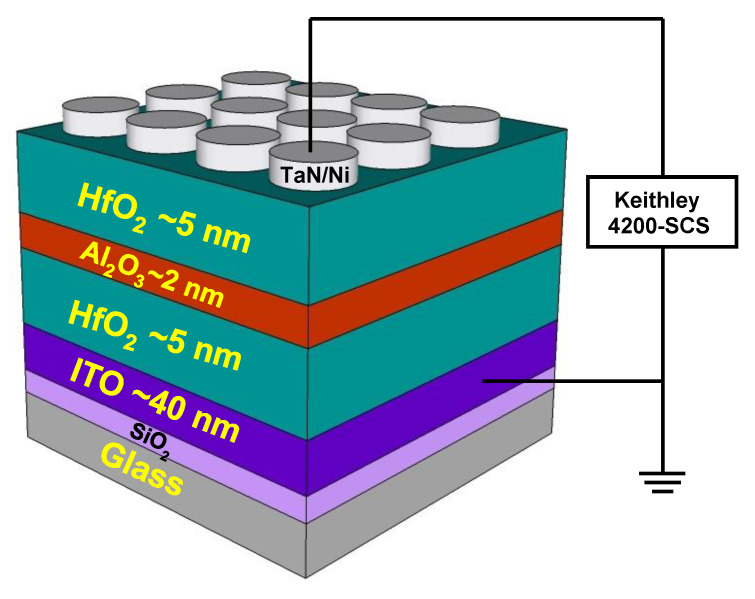
Schematic of TaN/HfO_2_/Al_2_O_3_/HfO_2_/indium tin oxide (ITO) resistive random access memory (RRAM) device structure with top and a bottom electrode connected to Keithley 4200 SCS semiconductor parameter.

Initially, the bottom electrode was taken as commercially available ~40-nm-thick ITO (sheet resistance of ~60 Ω/sq.) on SiO_2_/glass. Sequential ITO surface cleaning procedure was adopted stepwise with acetone, isopropyl alcohol, and deionized water along with ultrasonication for 5 min. Finally, it was dried using an N_2_ blow at room temperature. Cleaned ITO substrates were immediately transferred to the ALD system for Al_2_O_3_ and HfO_2_ deposition at low substrate temperature. Tri-layer of HfO_2_ (5 nm)/Al_2_O_3_ (2 nm)/HfO_2_ (5 nm) was deposited by using the metal precursors of tetrakis (ethylmethylamino) hafnium (TEMAH) and trimethylaluminum (TMA) for HfO_2_ and Al_2_O_3_, respectively. In this ALD technique, H_2_O was used as the oxidant at a substrate temperature of 150 °C. Sputtered TaN was used as a top electrode with Ni capping layer, and electrodes were formed by the liftoff process to achieve an area of 100 × 100 µm. Figure 1 shows the schematics of the fabricated tri-layer RRAM device. Electrical resistive switching (I‒V) and pulse measurements to characterize synaptic properties of the fabricated device, a Keithley 4200 SCS semiconductor parameter analyzer (Keithley Instrumnets, Cleveland, OH, USA), and a 4225-PMU ultrafast current-voltage (I–V) pulse module were used. All electrical measurements were obtained by applying a voltage to the top TaN electrode while the ITO bottom electrode (BE) was grounded. 

## 3. Results and Discussion 

**Figure 2:**
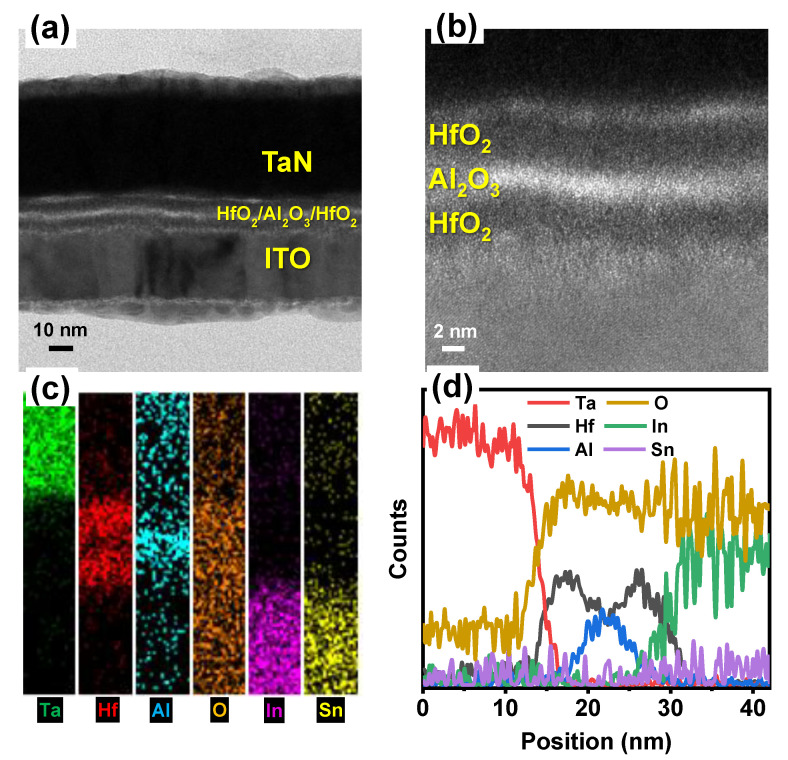
(**a**,**b**) Cross-sectional high-resolution transmission electron microscopy (HRTEM) image of TaN/HfO_2_/Al_2_O_3_/HfO_2_/ITO tri-layer RRAM structure; (**c**) EDS elemental mapping; (**d**) line profiles of Ta, Hf, Al, O, In, and Sn for the cross-section of the device.

**Figure 3:**
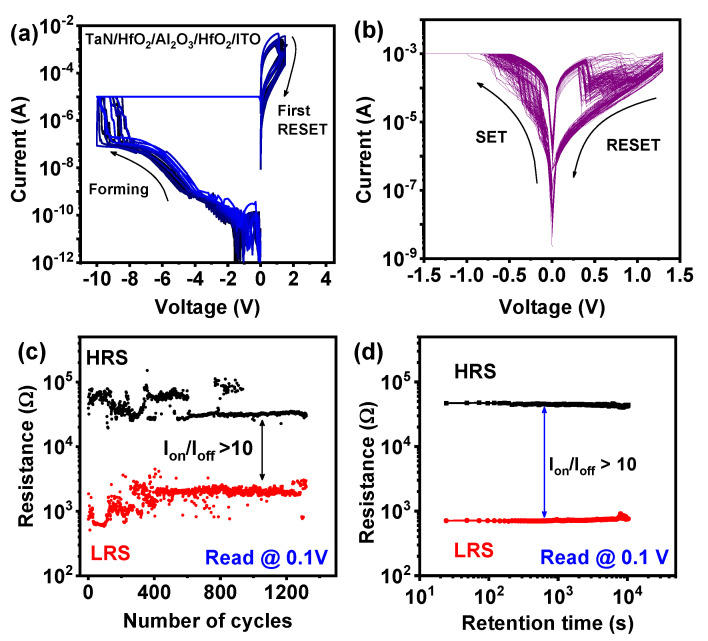
(**a**) Multiple initial forming characteristics under negative applied voltage to the top electrode and first RESET under positive voltage of TaN/HfO_2_/Al_2_O_3_/HfO_2_/ITO tri-layer RRAM device; (**b**) consecutive bipolar resistive switching characteristics of 200 SET/RESET cycles; (**c**) endurance characteristics of tri-layer RRAM device for 1300 switching cycles at a read voltage of 0.1 V; (**d**) memory retention of HRS and LRS for 10^4^ s with a read voltage of 0.1 V.

**Figure 4:**
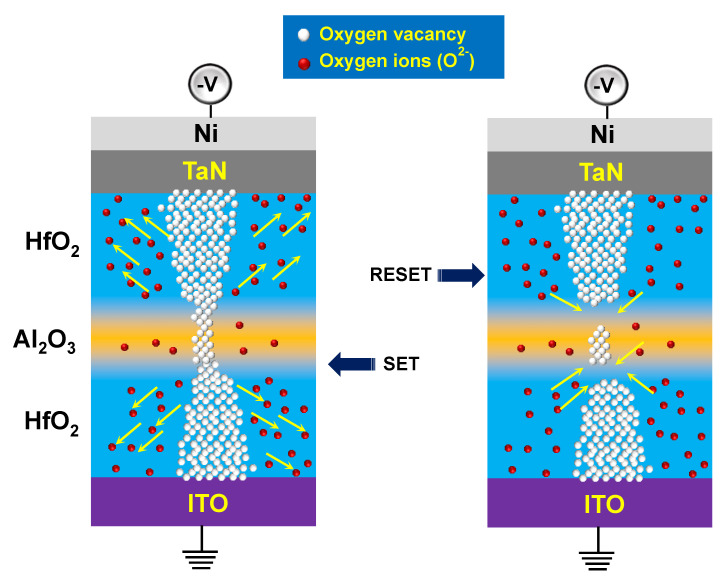
Schematic diagram of resistive switching mechanism of the TaN/HfO_2_/Al_2_O_3_/HfO_2_/ITO RRAM during the SET and RESET process.

The cross-section of the TaN/HfO_2_/Al_2_O_3_/HfO_2_/ITO tri-layer RRAM structure was investigated by the high-resolution transmission electron microscopy (HRTEM) image and the energy-dispersive X-ray spectroscopy (EDS) compositional mapping as shown in Figure 2. The total thickness of the tri-layer was confirmed to be ~12 nm, which was similar to the target thickness, as shown in Figure 2a,b. Different Ta, Hf, Al, O, In, and Sn element mapping confirms the presence of multilayer structures as shown in Figure 2c. To confirm the tri-layer more clearly, X-ray intensities of the line profiles are presented in Figure 2d, which confirmed the presence of HfO_2_/Al_2_O_3_/HfO_2_ tri-layer without any significant diffusion of In and Sn, which is due to low temperature (150 °C) ALD technique. In addition, from HRTEM and EDS analysis the inter-diffusion at Al_2_O_3_/HfO_2_ can be seen by the presence of hump, which can produce HfAlO, as presented in Figure 2d. According to Lan et al., intrinsic trap sites related to oxygen vacancies can be created due to the inter-diffusion between HfO_2_ and Al_2_O_3_ [30]. This inter-diffusion creates oxygen vacancies due to the presence of HfAlO in thin gate stacks. At both interface regions, the trivalent Al into HfO_2_ distributes intrinsic oxygen vacancies (V_O_), which control the gradual resistive switching in the RRAM device discussed in the next section. Figure 3a shows the forming and first RESET characteristics of the TaN/HfO_2_/Al_2_O_3_/HfO_2_/ITO tri-layer RRAM device. The initial forming process shows the similar behavior of multiple devices for the I-V soft breakdown under negative voltage applied to the top TaN electrode where the initial current compliance was set to 10^−5^ A. During the electroforming process, the conductive filament forms between two electrodes, which consist of mainly oxygen vacancies (V_O_). Gradual SET/RESET bipolar switching properties were found after the electroforming process at the SET current compliance of 10^−3^ A. Gradual SET/RESET during resistive switching is the requirement for future synaptic device applications for neuromorphic computing [31]. During the SET process, V_O_ regrowth inside both the interfacial layers of Al_2_O_3_/HfO_2_ helps gradual change occur in the current, as shown in Figure 2b. Here, both HfO_2_ layers act as a virtual electrode (as HfO_2_ contains more oxygen vacancy compared to Al_2_O_3_) and during the RESET process only interface filament is believed to rapture due to positive bias at the top electrode, which controls the resistive switching process stability and reliability [20]. A schematic resistive switching mechanism has been presented in Figure 4. 

After applying a negative bias at the electrode, oxygen vacancies are piled up mainly near the interface driven by the external electric field. The virtual electrode (formed during electroforming process) at both ends within HfO_2_ helps to regrow the CF. During RESET process, the CF is not fully ruptured, and V_O_ recombines with oxygen ions gradually due to the applied positive bias at the top electrode, as shown in Figure 4. According to previous studies, asymmetric CF forms inside two different dielectrics. This further leads to formation of the weakest CF at the interface of two different dielectrics [14,32]. Hence, the redox reaction due to the migration of oxygen ions dominates the RESET process near the interface of two dielectrics, as described, with the switching mechanism in Figure 4 [20,32].The endurance characteristics were obtained for the tri-layer RRAM device with up to 1300 cycles with I_on_/I_off_ ratio > 10, as shown in Figure 3c, read at 0.1 V. Initial variation of low resistance state (LRS) and high resistance state (HRS) can be due to the large area of the top electrode, where a large number of conductive filaments are created during the forming process. Data retention at a read voltage of 0.1 V, for LRS and HRS, was recorded up to 10^4^ s without any significant variation, as shown in Figure 3d. To understand the performance of the proposed tri-layer RRAM device, a detailed comparison of electrical parameters is presented in Table 1. 

**Figure 5:**
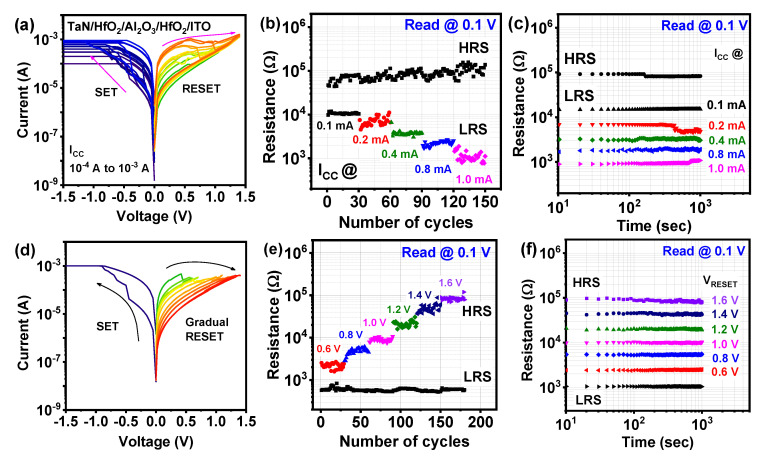
(**a**) Multilevel storage of tri-layer RRAM device under varying SET compliance currents from 100 µA to 1 mA; (**b**) reliability test for data endurance (30 cycles); (**c**) retention characteristics (10^3^ s) with the corresponding increasing SET I_cc_; (**d**) bipolar resistive switching with multilevel high resistance state under increasing RESET voltage for tri-layer memristor device; (**e**) multi-level endurance characteristics under different RESET voltages for 30 cycles each; (**f**) retention characteristics of LRS and multilevel HRS under different RESET V_stop_ for 10^3^ s.

Multi-level resistance states depending on the SET current compliance (I_cc_) were investigated to find the influence of increasing oxygen vacancy concentration inside conducting filaments (CF) [36,37,38]. Figure 5a shows the resistive switching characteristics with variable I_cc_ during the SET process from 100 µA to 1 mA. From low to high I_cc_, the resistance of the device continuously decreased as shown in Figure 5a. For the endurance test of each LRS, 30 cycles of each resistance state were monitored. As evident from Figure 5b, HRS remains almost constant and multiple LRS was found to be with 10.54 kΩ, 6.08 kΩ, 3.57 kΩ, 2.13 kΩ, 1.01 kΩ at the read voltage of 0.1 V. The decrease in resistance can be explained by the increased width of CF inside tri-layer dielectric films, concerning the continuous enhancement of V_O_ [36]. The stability of different resistance states was confirmed by the retention test, as shown in Figure 5c, where different LRS maintained up to 10^3^ s without any significant variation. Modulation of a multi-level conduction state is a very essential aspect for the synaptic device to realize the high-density memory storage. To achieve multilevel memory states, gradual RESET has been controlled by V_reset-stop_, as shown in Figure 5d. A similar approach has been shown in recent works on bilayer and tri-layer RRAM structures to obtain multi-state resistance by controlling V_reset-stop_ [21,33,39,40]. Here, the positive RESET voltage was slowly increased to get a gradual RESET process in the HfO_2_/Al_2_O_3_/HfO_2_ tri-layer RRAM device, which leads to multiple HRS.

As mentioned above, the filament dissolution increased continuously at both Al_2_O_3_/HfO_2_ interface under increasing positive top electrode bias, which further leads to changing the device’s resistance. The stability of different HRS was confirmed by endurance tests for 30 cycles of each V_reset-stop,_ as shown in Figure 5e. As shown in Figure 4e, LRS along with multiple HRS were obtained from the RESET I-V read at 0.1 V. Different average resistance states were found to be 2.26 kΩ, 5.02 kΩ, 9.07 kΩ, 20.5 kΩ, 47.2 kΩ, 87.3 kΩ at the RESET voltage of 0.6 V, 0.8 V, 1.0 V, 1.2 V, 1.4 V, 1.6 V, respectively. Data retention is another important property for different HRS stabilities and synaptic applications. Figure 5f shows the retention properties up to 10^3^ s for distinct LRS and six HRS states for testing the reliability of multi-state resistance. From this above experiment, it was confirmed that the HfO_2_/Al_2_O_3_/HfO_2_ tri-layer is suitable for high storage and multi-level analog RRAM applications.

**Figure 6:**
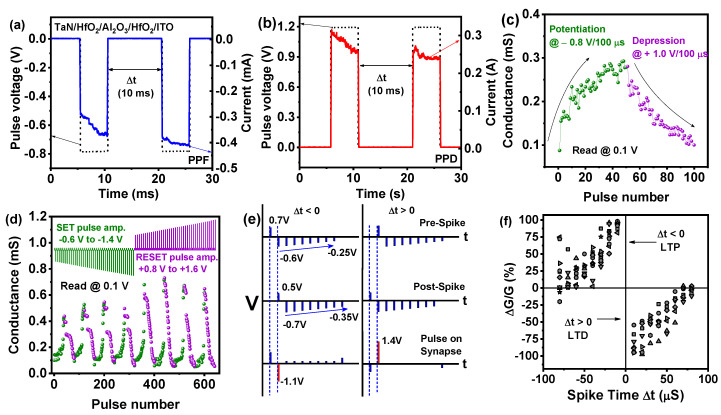
(**a**) Paired-pulsed facilitation and (**b**) paired-pulsed depression phenomenon of tri-layer TaN/HfO_2_/Al_2_O_3_/HfO_2_/ITO memristor device under pulse spikes of −0.8 V and +1.2 V, respectively, with a time interval of 10 ms; (**c**) pulse potentiation/depression cycle with constant pulse width of −0.8 V/100 µs and +1.0 V/100 µs, respectively, read at 0.1 V (50 pulses of potentiation and 50 pulses of depression). (**d**) Repetitive 8 cycles of gradual conductance modulation for consecutive potentiation and depression controlled by increasing step pulse and read at 0.1 V; (**e**) details of pre-pulse and post-pulse scheme design using time-division multiplexing (TDM) approach to realize spike-timing-dependent plasticity (STDP) properties; (**f**) STDP characteristics of tri-layer RRAM device shows relative synaptic weight (ΔW) change with respect to spiking timing (Δt).

Along with gradual conductance change under DC voltage, modulation of resistance was also studied by a sequential paired pulse. Analogous to the bio-synapse, suitable paired pulse application can change the resistance of the RRAM devices with a short interval of time [41,42,43]. So, it is believed that synapse response is higher at the second pulse if a paired pulse is applied to the synapse [44]. Figure 6a,b show the pair-pulse fluctuation (PPF) and paired-pulse depression (PPD) characteristics for TaN/HfO_2_/Al_2_O_3_/HfO_2_/ITO memristor device. PPF and PPD responses are monitored after implementing paired pulse of −0.8 V/5 ms and +1.2 V/5 ms, respectively, with an interval of 10 ms, which is the short-term change in synaptic weight. In the case of PPF, an increase in current can be noticed at the response of the second pulse compared to the first pulse, which indicates the generation of V_O_. In the case of PPD, the opposite phenomenon took place, where the pulse current was found to be reduced due to recombination of oxygen vacancies and oxygen ions. Therefore, this result indicates that tri-layer RRAM can simulate the bio-synapse in real-time signals. Calculated average PPF and PPD was calculated to be ~10.2% and ~7.5%, respectively, from the equation,

PPF = (I_2_ − I_1_)/I_1_ × 100% (1)

where I_1_ and I_2_ are the final currents recorded at each paired pulse [45]. 

Adjustable gradual conductance increase and decrease (potentiation/depression) are very essential for electronic synapse and have been studied in this section. The tri-layer RRAM device exhibits a gradual change in conductance after applying a constant amplitude pulse train of −0.8 V/100 µs and +1.0 V/100 µs for potentiation and depression, respectively, as shown in Figure 6c. A consecutive 50 cycles of negative pulses and 50 cycles of positive pulses were applied to achieve gradual conductance change, which is consistent with the DC gradual switching behavior discussed before. These properties indicate the synaptic plasticity in response to the pulse train, similar to the long-term potentiation (LTP), and long-term depression (LTD) in biological synapse [46]. This conductance change phenomenon is assumed to be dominated by the separation and recombination of oxygen vacancies (V_O_) and oxygen ions near both interfaces of HfO_2_/Al_2_O_3_ after applying different polarity pulse at the top electrode. During RESET, oxygen vacancies and oxygen ions recombine slowly and reduce the filament width at the weak filaments formed at both HfO_2_/Al_2_O_3_ due to the application of positive pulse train [25]. During the application of the negative pulse train, again gradually the weak filaments formed at both interfaces. To implement more accurately synaptic efficiency, of the tri-layer TaN/HfO_2_/Al_2_O_3_/HfO_2_/ITO memristor, negative pulse, and positive depression pulse with increasing amplitude were applied for potentiation and depression characteristics [47,48,49]. For potentiation and depression, increasing the pulse amplitude from −0.6 to −1.4 V, with a −0.05 V step, and 0.8 to 1.6 V, with a 0.05 V step, respectively, was applied to each tri-layer RRAM device. The applied pulse sequence is presented in Figure 6c, with a read voltage of 0.1 V. A clear gradual increase/decrease in conductance was observed during the long-term potentiation (LTP) and long-term depression (LTD) process, which is similar to the synaptic change in the biological synapse. The change in conductance was measured by the peak current obtained from the read pulse of 0.1 V as shown in Figure 6d. An almost gradual increase in conductance and a gradual decrease in conductance was observed during all 8 cycles. During potentiation at increasing negative pulse voltage to the top electrode oxygen ions were depleted mainly from the HfO_2_/Al_2_O_3_ interface and created weak CF. The opposite phenomenon occurs with positive increasing pulse voltage at the top electrode and gradually the CF at the HfO_2_/Al_2_O_3_ interface becomes narrow. Successful implementation of eight cycles of potentiation and depression is presented, which proves the reliability of the synaptic property of the tri-layer device. Slightly increasing conductance behavior at LTP can be due to the occurrence of new V_O_ creation at the HfO_2_/Al_2_O_3_ interface. 

To emulate the Hebbian learning of spiking neural networks (SNN), we focus on mimicking the spike-timing-dependent plasticity (STDP) learning rule in the tri-layer TaN/HfO_2_/Al_2_O_3_/HfO_2_/ITO memristor, having been employed to simulate synapse functionality [50]. This learning rule depends on the relative time difference (Δt) of a set of spikes related to the pre-synaptic and post-synaptic neurons [51]. Design of the pre-spike, post-spike and consequent effective pulse applied to the synapse for time-division multiplexing (TDM) approach are shown in Figure 6e. Using this pulse sequence, the obtained STDP characteristics for synaptic learning rules in the tri-layer RRAM device was employed using the TDM approach, as shown in Figure 6d [50]. Presynaptic spikes and postsynaptic spikes were included as +0.7, −0.6, −0.55, −0.5, −0.45, −0.4, −0.35, −0.3, and −0.25 V and +0.5, −0.7, −0.65, −0.6, −0.55, −0.5, −0.45, −0.4, and −0.35 V, respectively. Here, it is considered that when the pre-spike precedes the post-spike (Δt > 0), potentiation occurs, and in the opposite case when the post-spike precedes the pre-spike (Δt < 0) the device is depressed. Positive synaptic weight change is observed in the IInd quadrant when Δt increases from 0 to ‒100 µs, which indicates synaptic potentiation, and negative synaptic weight change observed in the IVth quadrant when Δt varies from 0 to 100 µs is described as synaptic depression. The change of synaptic weight can be described as follows:

(2) $$\Delta w=\{\begin{array}{c} A_{+}e^{-\Delta t/\tau_{+}}\\ -A_{-}e^{-\Delta t/\tau_{-}} \end{array}\begin{array}{c} \Delta t>0\\ \Delta t<0 \end{array}$$

where A_+_ and A_‒_ are the synapse maximum weights at Δt = 0, and τ_+_ and τ_‒_ are the broadening of the STDP window [48]. From Figure 6d, it can be found that the maximum change in synaptic weight [ΔW(ΔG/G) = (G_fin_ − G_min_)/G_min_] for positive Δt was 98.3% and for negative Δt was −94.8%, which is very symmetric for tri-layer RRAM device. These results further confirm the superiority of the stable and reliable synaptic characteristics of the TaN/HfO_2_/Al_2_O_3_/HfO_2_/ITO memristor device.

## 4. Conclusions

In summary, bipolar resistive switching behavior and synaptic properties of ALD deposited TaN/HfO_2_/Al_2_O_3_/HfO_2_/ITO memristor were studied in detail. Bipolar resistive switching with a gradual change in conductance was confirmed by DC I-V and pulse application. Uniform gradual resistive switching can be attributed to the formation of HfAlO in the interface of HfO_2_/Al_2_O_3_ in the tri-layer dielectric stack, where more than 10^3^ cycles of endurance and 10^4^ s of retention were achieved. Through applying different pulse sequences, short-term plasticity and symmetrical long-term plasticity were studied by PPF and potentiation/depression. Successful STDP behavior was achieved using the TDM method with conductance change from −94.8% to 98.3%. The above results for tri-layer RRAM predict a promising nonvolatile memory based synaptic device for the next generation. Although optimization of deposition parameters and thickness of the dielectric stacks are needed in the future.

## Figures and Tables

**Table 1:** Comparison of electrical parameters for different RRAM structures.

RRAM Structure	Operation Mode	Forming Voltage (V)	HRS/LRS Ratio	Endurance (Cycles)	Retention (s)	I_cc_ (A)	V_set_ (V)	V_reset_ (V)	Ref.
AlO_X_/HfO_X_	Bipolar	NA	10^2^	400	10^4^	10^−3^	0.8	−1.0	[33]
ZrO_2_/HfO_2_	Bipolar	−4	10^2^	10^7^	10^4^	10^−3^	1.0	−1.0	[14]
Al_2_O_3_/HfO_2_	Bipolar	NA	>10	500	NA	10^−3^	0.9	−0.8	[12]
Hf_x_Al_y_O	Bipolar	2.7	10^2^	10^4^	10^4^	10^−3^	0.6	−0.5	[34]
HfO_2_/Al_2_O_3_	Bipolar	5.2	10^2^	10^4^	10^4^	10^−5^	1.8	−1.5	[4]
Cu_2_O/Al_2_O_3_	Bipolar	−3.0	10^2^	NA	NA	10^−4^	−1.7	1.0	[35]
HfO_2_/TiO_x_	Bipolar	3.7	10^2^	10^4^	NA	10^−6^	1.5	−1.1	[17]
TiO_2−x_/HfO_2−y_/TiO_2−x_	Bipolar	NA	10^2^	10^7^	10^5^	free	5.0	−5.0	[10]
Al_2_O_3_/HfO_2_/Al_2_O_3_	Bipolar	NA	10^2^	10^5^	10^5^	free	−1.0	1.5	[21]
HfO_2_/TiO_2_/HfO_2_	Bipolar	−3.7	10^2^	200	10^4^	10^−3^	−1.0	1.5	[11]
ZrO_2_/ZrO_2−x_/ZrO_2_	Bipolar	NA	>10	100	NA	free	−5.0	6.0	[24]
Al_2_O_3_/HfO_2_/Al_2_O_3_	Bipolar	−2.0	>10	10^3^	10^4^	10^−2^	−1.0	1.25	[20]
HfO_2_/Al_2_O_3_/HfO_2_	Bipolar	−9.0	>10	>10^3^	10^4^	10^−3^	−0.3	0.8	This work
